# Extraction of oil from wet Antarctic krill (*Euphausia superba*) using a subcritical dimethyl ether method

**DOI:** 10.1039/c9ra06238f

**Published:** 2019-10-24

**Authors:** Shulai Liu, Wei Hu, Yizhou Fang, Yanping Cai, Jianyou Zhang, Jianhua Liu, Yuting Ding

**Affiliations:** Department of Food Science and Engineering, Ocean College, Zhejiang University of Technology Hangzhou 310014 P. R. China jhliu@zjut.edu.cn dingyt@zjut.edu.cn +86 571 88320237 +86 571 88320237; National R & D Branch Center for Pelagic Aquatic Products Processing (Hangzhou) Hangzhou 310014 P. R. China; Collaborative Innovation Center of Seafood Deep Processing, Dalian Polytechnic University P. R. China; Key Laboratory of Marine Food Quality and Hazard Controlling Technology of Zhejiang Province, College of Life Sciences, China Jiliang University Hangzhou Zhejiang 310018 P. R. China

## Abstract

In this study, a novel method for obtaining high-quality krill oil from wet Antarctic krill by using subcritical dimethyl ether (SDE) was proposed. A response surface design was used to obtain the best SDE extraction parameters. The optimum extraction efficiency of 93.77 ± 0.92% was obtained at a stirring speed of 1030 rpm, temperature of 47 °C and dynamic extraction time of 90 min. Compared with *n*-hexane, ethanol, supercritical CO_2_ and subcritical *n*-butane extraction, the krill oil extracted by SDE exhibited low peroxide values (1.46 ± 0.26 mmol kg^−1^), high astaxanthin (218.06 ± 4.74 mg kg^−1^), phosphatidylcholine (PC) (33.95 ± 0.65%), and phosphatidylethanolamine (PE) (11.67 ± 0.23%) content. Moreover, krill oil extracted by SDE has high levels of EPA (16.38 ± 0.05%) and DHA (7.91 ± 0.07%). SDE extraction proved to be an efficient and safe method for extraction of quality krill oil from wet Antarctic krill, and it could be a promising method for oil extraction in wet food in future.

## Abstract 2

In this study, a novel method for obtaining high-quality krill oil from wet Antarctic krill by using subcritical dimethyl ether (SDE) was proposed.

## Graphical abstract

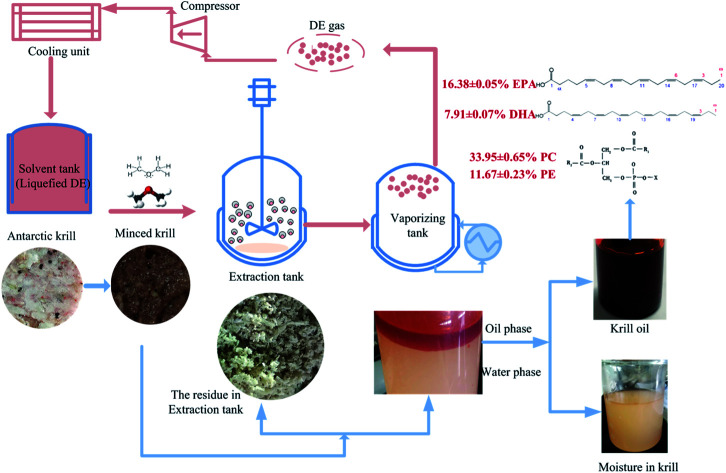

## 1. Introduction

Antarctic krill, one of the largest of eighty-six krill species, mainly inhabit the Southern Ocean.^1^ Antarctic krill are small, shrimp-like invertebrate zoology and are generally distributed in aggregations or swarms. Many scientists assume that krill are one of the most abundant biomass species on the planet, with a preliminarily estimated total biomass of around 500 million metric tons.^2^

According to Grantham, the main composition of Antarctic krill is protein (11.9–15.4%), lipid (0.5–3.6%), ash (3%), carbohydrates (2%), and water (77.9–83.1%).^3^ The lipid in Antarctic krill is one of the richest sources of *n*-3 polyunsaturated fatty acids (PUFAs), which contain abundant eicosapentaenoic acid (EPA) and docosahexaenoic acid (DHA).^4,5^ Numerous research studies have proved that *n*-3 PUFAs have positive effects on improving physical health and reduce risk of diseases.^6^ For example, they could reduce risk factors for cardiovascular diseases, decrease inflammatory diseases, improve immune function and decrease tumour cell growth and survival.^6–8^ Therefore, krill oil can be considered a potential source of lipid with unique biological efficacy.^9^ Additionally, EPA and DHA in krill oil are mainly bound to phospholipids (PL) rather than triglyceride as compared to fish oil. Numerous scientists have reported that the *n*-3 PUFAs attached with PL are more efficiently absorbed and incorporated into cell membranes than TAG,^10,11^ which means that *n*-3 PUFAs of krill oil has better bioavailability. Therefore, krill oil might be expected to be a superior source for omega-3 market.

However, the research about the extraction technology for Antarctic krill oil has not made much progress. At present, the common methods for extracting krill oil from krill meal mainly include organic solvent extraction, supercritical fluid extraction and subcritical fluid extraction. According to the principle of similar compatibility, different polar solvents can be used for obtaining lipids from krill meal.^12–15^ However, several drawbacks could be mentioned for conventional solvent extraction, such as high consumption for solvents, toxic solvents residual. The supercritical CO_2_ extraction has been applied to heat-sensitive bioactive compounds extraction. However, it has some drawbacks as follows: (1) CO_2_ has lower solubility of polar lipids because of its non-polar nature;^16,17^ (2) higher operation cost. In recent years, subcritical fluid extraction has been greatly developed as a novel technology, mainly including subcritical water, propane, *n*-butane and dimethyl ether. To the best of our knowledge, the extraction of Antarctic krill lipid by subcritical *n*-butane has been reported.^12,18^ But subcritical *n*-butane was not a suitable method for extracting polar lipid from Antarctic krill due to the weaker polarity of butane. Moreover, like the above-mentioned conventional methods, subcritical *n*-butane was suitable for extracting krill oil from dried krill meal. The krill meal produced by traditional heat drying methods would destroy the bioactive compounds and deteriorate the quality of krill oil. And the freeze-drying process would undoubtedly increase the overall cost. Therefore, there was an urgent need in the market for a novel krill oil extraction technology to overcome these processing challenges.

In recent years, SDE extraction as a promising “green” technique has attracted tremendous attention. This might be attributed to the following advantages of DE: (1) low normal boiling point (−24.8 °C) suggesting lower solvent residual;^19^ (2) DE has high affinity to oily substances and partial miscibility with water;^16^ (3) DE exhibiting non-toxicity in lipid extraction has been considered as a green and safe solvent for food industry by the European Food Safety Authority.^20,21^ Thus far, there have been many studies on SDE applications: (1) extraction of water from high-moisture coal;^22^ (2) removal of undesired contaminants from polluted materials;^23,24^ (3) extraction of lipids or bioactive components from high-moisture materials. For example, extraction lipids from high-moisture tuna liver^25^ or microalgae^19,26^ and extraction of free lutein from marigold flowers.^27^ In a word, SDE was an efficient, safe, reliable, environmentally friendly and green sustainable technique. It could be particularly useful for extraction of polar and neutral lipids from high-moisture raw materials.

In this work, the feasibility of extraction of polar krill oil from the high moisture Antarctic krill was investigated. Moreover, the extraction parameters (stirring speed, extraction temperature and time) of SDE were also optimized for the optimal extraction efficiency of oil using response surface methodology (RSM). The chemical properties and bioactive components of the krill oil obtained by SDE was comprehensively analyzed and evaluated as compared to those obtained by *n*-hexane, ethanol, supercritical CO_2_, subcritical *n*-butane extraction. These results would be beneficial for providing powerful insights into the feasibility of extraction of krill oil from wet Antarctic krill using SDE.

## 2. Materials and methods

### 2.1. Materials

Antarctic krill (moisture content, 78.90 ± 0.75%) was supplied by Liaoyu Group Co., Ltd. (Dalian, China) and frozen at −23 °C. A part of them were used to prepare krill powder by a vacuum freeze dryer (Scientz-12N, Ningbo Science Biotechnology Co. Ltd, Ningbo, China) for 48 h and stored at −23 °C. The other part of them were used for extraction krill oil by SDE after thawed.

The DE (purity > 99.9%) was purchased from Hangzhou Airco Refrigerant Technology Co., Ltd. (Hangzhou, china). The standards of 37 fatty acid methyl esters (FAMEs), astaxanthin (≥97% HPLC), l-α-phosphatidylcholine (PC), l-α-phosphatidylethanolamine (PE), tocopherol and retinol were purchased from Sigma-Aldrich (Shanghai, China). All other reagents and solvents were provided by Fisher Scientific (Shanghai, China).

### 2.2. Extraction of krill oil

#### 2.2.1. SDE extraction

**Fig. 1:**
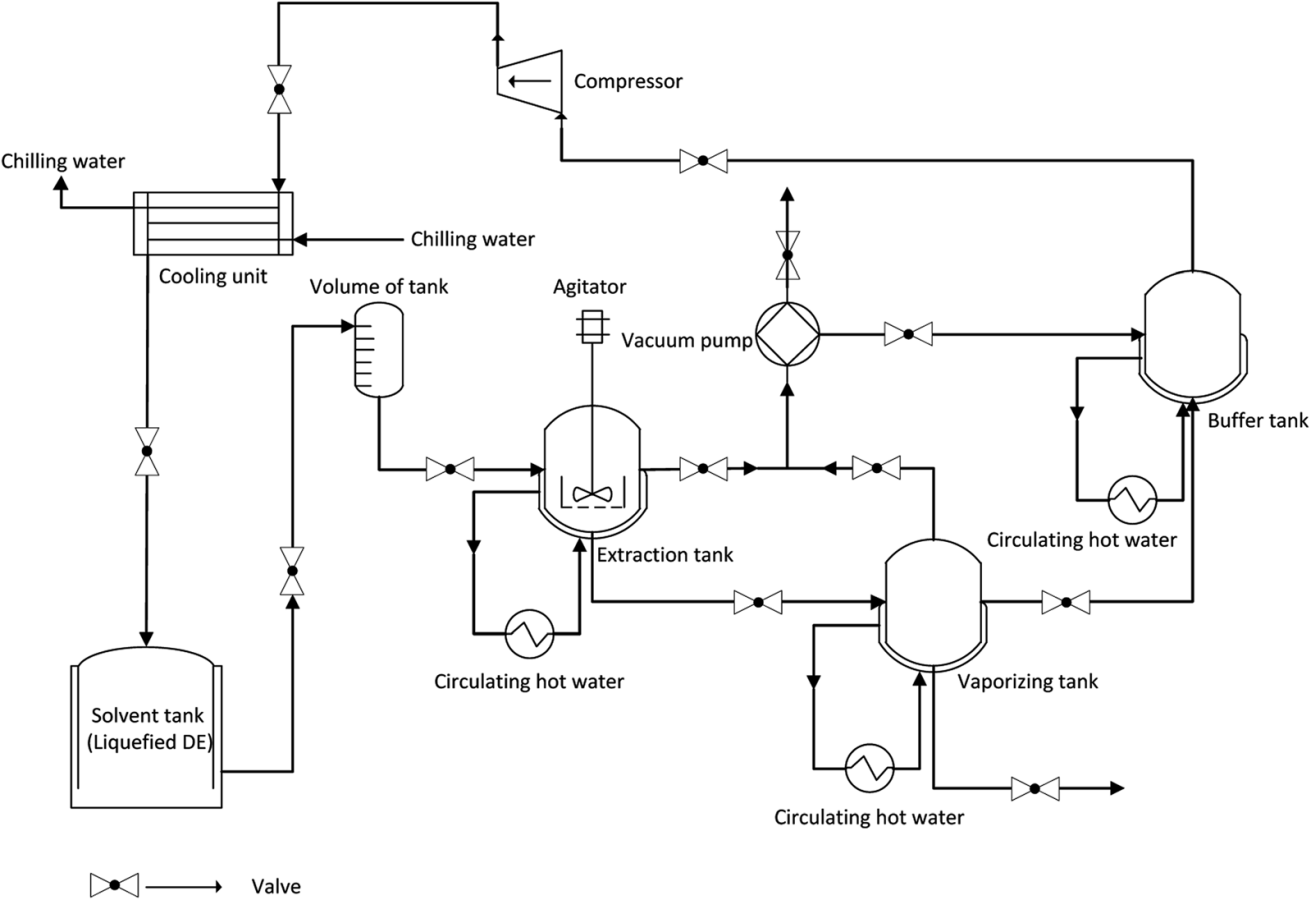
Schematic diagram depicting subcritical dimethyl ether extraction.

The subcritical fluid extraction system shown in Fig. 1 (CBE-5L, Henan Subcritical Biological Technology Co., Ltd., Anyang, China) was used for extracting krill oil in this study. Furthermore, subcritical fluid extraction apparatus and operational program were described in detail in our previous study.^25^

Firstly, thawed krill was minced by mincing machine before SDE extraction. Secondly, samples of 150 g krill and DE (20 mL g^−1^ sample) were placed into the extraction vessel with filters. After extraction, DE was evaporated (30 °C) in order to gather the extracted oil. Then *n*-hexane (20 mL) was used to wash the evaporation tank to ensure the complete collection of the residual oil that sticked to the wall of the tank. The water and *n*-hexane layer were separated by centrifugation at 8000 rpm. Finally, the *n*-hexane was removed by rotary vacuum evaporator at 45 °C to obtain krill oil. On the basis of single factor experiment, stirring speed (*X*_1_, rpm), dynamic extraction time (*X*_2_, min) and extraction temperature (*X*_3_, °C) were determined as key variables. The three-variable-three-level Box–Behnken design (BBD, software Design-Expert.V8.0.6, Stat-Ease, Inc, Minneapolis, U.S.) was applied to optimize the extraction efficiency. Table 1 details the BBD matrix and corresponding experimental results. All experiments were performed in a randomized order.

**Table 1:** Box–Behnken experimental design matrix (actual and coded) and results of extraction efficiency for krill oil

Run	*X*_1_	*X*_2_	*X*_3_	*Y*
1	1000 (0)	60 (−1)	30 (−1)	84.92
2	1250 (+1)	80 (0)	30 (−1)	87.86
3	1000 (0)	80 (0)	40 (0)	92.19
4	1250 (+1)	80 (0)	50 (+1)	93.49
5	1000 (0)	80 (0)	40 (0)	92.57
6	1000 (0)	80 (0)	40 (0)	92.95
7	750 (−1)	80 (0)	50 (+1)	92.57
8	1000 (0)	100 (+1)	30 (−1)	88.58
9	1000 (0)	80 (0)	40 (0)	92.04
10	750 (−1)	60 (−1)	40 (0)	88.43
11	1000 (0)	80 (0)	40 (0)	92.22
12	1000 (0)	60 (−1)	50 (+1)	92.13
13	1250 (+1)	100 (+1)	40 (0)	93.31
14	1000 (0)	100 (+1)	50 (+1)	94.11
15	750 (−1)	100 (+1)	40 (0)	91.55
16	750 (−1)	80 (0)	30 (−1)	85.98
17	1250 (+1)	60 (−1)	40 (0)	91.1

#### 2.2.2. Subcritical *n*-butane extraction

According to the specific conditions for extracting krill oil by subcritical *n*-butane described by Sun, *et al.*,^18^ the detailed extraction conditions were as follows with slight modifications: ratio of krill powder to *n*-butane 1 : 20 (w/v), dynamic extraction time 120 min, extraction temperature 50 °C.

#### 2.2.3. Conventional solvent extraction

Because of the low toxicity of ethanol and *n*-hexane, they were usually selected for extraction of Antarctic krill oil. Sample of 100 g krill powder and 1200 mL solvent were added to extraction vessel equipped with stirrer, and the mixture was transferred to water bath (40 °C) and extracted for 120 min. Subsequently, the krill oil was recovered using rotary vacuum evaporator at 45 °C.

#### 2.2.4. Supercritical CO_2_ extraction

The krill oil extraction was performed by supercritical CO_2_ extraction apparatus (SFE-Prime, Applied Separation, Allentown, America). The extraction condition was described by Ali-Nehari, Kim, Lee, Lee, and Chun^28^ with slight modifications. A certain amount of krill powder (15 g) was used for each experimental. During extraction process, the supercritical CO_2_ extraction apparatus was controlled at 30 MPa, 45 °C and 120 min, with a continuous CO_2_ flow of 3 mL min^−1^. After extraction, krill oil was recovered, weighed and used for the next analyses.

### 2.3. Calculation of lipid extraction efficiency

For each extraction method, the weight of initial krill prior to extraction was recorded. After extraction, the weight of recovered krill oil was recorded, and the total lipid content of krill by Folch method (Floch)^29^ was determined to calculate the extraction efficiency.
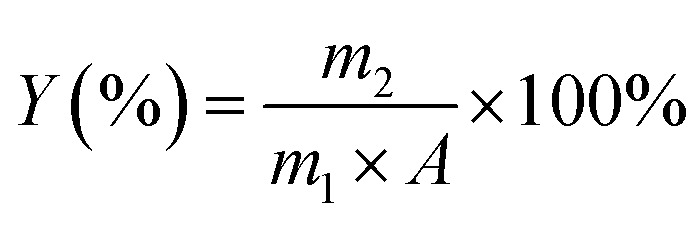
where *Y* is the extraction efficiency; *m*_2_ (g) is the weight of the recovered krill oil; *m*_1_ (g) is the weight of initial raw material and *A* (g/100 g) is the total lipids content of krill.

### 2.4. Measurement for chemical properties of krill oil

The acid values (Cd 3d-63), peroxide values (Cd 8-53), iodine values (Cd 1-25) and non-saponification matter (Ca 6b-53) of krill oil were performed according to the method described by the American Oil Chemists' Society.^30^

### 2.5. Determination of PC and PE for krill oil

The PC and PE contents were measured by high-performance liquid chromatographic system (HPLC) (E2695, Waters, Massachusetts, America) equipped with a 2998 Photodiode Array detector (PAD) (Waters, Massachusetts, America). According to the method described by Wang, *et al.*^31^ with slight modifications. Briefly, an aliquot of 0.1 g of krill oil was dissolved in 50 mL *n*-hexane/2-propanol (2 : 3) solvent and 10 mL of the solution were used for HPLC-PAD analysis. The sample separation was performed on a silica column (5 μm, 4.6 mm × 250 mm; Waters, Massachusetts, America) using *n*-hexane/2-propanol/0.05% acetic acid (45 : 50 : 5, v/v) as the mobile phase with a rate of 1.0 mL min^−1^. The determined wavelengths were identified 206 nm for PC and PE, respectively. The PC and PE of samples were identified and quantified by comparing with standards.

### 2.6. Fatty acids composition of krill oil

The preparation of fatty acid methyl esters for krill oil using KOH–methanol esterification was based on the method described by Yin, *et al.*^32^ with slight modifications. Briefly, an aliquot of 50 mg of lipid sample was transferred into screw capped test tube and mixed with 0.5 mol mL^−1^ KOH–methanol solution (2 mL). Then tightly capped, the test tube was placed into water bath at 80 °C for 10 min, shaken for two times every minute. After cooling quickly to room temperature in cold water bath, 14% boron trifluoride–methanol solution (2 mL) was added to screw capped test tube and heated at 80 °C for 5 min in water bath with continuous shaking. The fatty acid methyl esters were collected with 5 mL *n*-hexane and then centrifugation for 5 min at 8000 rpm. The upper layer was filtered by 0.22 μm filter membrane and used for next analysis.

The fatty acid methyl esters were analyzed by a TRACE 1300 (Thermo Fisher Scientific, Franklin, MA, America) equipped with a HP-INNOWax MS capillary column (60 m × 0.32 mm × 0.25 μm; Agilent Technologies Inc., Santa Clara, CA, America). Helium was used as the carrier gas, with constant current mode, and flow rate 2 mL min^−1^. The column temperature profile was the initial temperature of 90 °C maintained for 5 min, raised to 200 °C at a rate of 15 °C min^−1^, raised to 240 °C at a rate of 1 °C min^−1^ and kept at 240 °C for 10 min. The injection volume was 1 μL with no split. The MS was in EI mode (70 eV) with a 0.2 scan per s, interval over a 35–450 *m*/*z* range. Most of the fatty acid methyl esters were identified and quantitated by comparison of retention time and relative percentages areas of known standards of 37 FAME mix.

### 2.7. Determination of astaxanthin content for krill oil

The astaxanthin content was determined by a HPLC (E2695, Waters, Massachusetts, America) equipped with a PAD (2998, Waters, Massachusetts, America). The saponification of astaxanthin esters was performed according to the method by Xie, *et al.*^13^ The final product was separated on a C18 column (5 μm, 4.6 mm × 250 mm; Thermo Fisher Scientific, Franklin, MA, USA, America) using methanol, dichloromethane, acetonitrile and water (85 : 5 : 5 : 5) as mobile phase at a flow rate of 1 mL min^−1^. The determined wavelength was 476 nm for astaxanthin, and the content of astaxanthin for samples was quantified by comparing the peak area of the standard astaxanthin.

### 2.8. Determination of tocopherols and vitamin A for krill oil

The tocopherols and vitamin A content in krill oil was determined according to the method as described by Fang, Liu, Hu, Zhang, Ding and Liu^33^ with some modifications. Briefly, approximately 2 g krill oil were weighed into boiling flask and mixed with 0.5 g of ascorbic acid, 40 mL of ethanol and 15 mL of KOH (50%). Subsequently, the mixed solution was saponified at 80 °C for 30 min to ensure complete saponification of tocopherol ester and retinol ester, and 20 mL *n*-hexane was used to extract the non-saponification maters three times. After *n*-hexane layer was washed with deionized water until neutralization, the residue was obtained by removing *n*-hexane. Then the residue was dissolved in 10 mL of methanol. Ten microliters of the product was injected to a HPLC (Waters, Massachusetts, America)-PAD (2998, Waters, Massachusetts, America) and the separation was performed on a C18 column (5 μm, 4.6 mm × 250 mm; Thermo Fisher Scientific, Franklin, MA, USA, America) by controlling the gradient flow rate 1.0 mL min^−1^ of the mobile phase (methanol : water = 96 : 4). Readings of the tocopherols and vitamin A were then taken at 294 nm and 325 nm, respectively. The α-tocopherols and vitamin A of samples were identified and quantified by comparing them with the standards.

### 2.9. Statistical analysis

Statistical analysis was performed using IBM SPSS statistics software (v. 19.0, SPSS, Inc., Chicago, IL). Each experiment was performed repetition at three times in order to ensure their reproducibility and the results were expressed as the form of mean ± SD. Statistical comparisons were processed by one-way analysis of variance (ANOVA) using Tukey's test with a 0.05-level of significance.

## 3. Results and discussion

### 3.1. Response surface optimization of SDE extraction condition

The response surface methodology with Box–Behnken design (BBD) was used to optimize the SDE extraction parameters. The detailed experimental combinations and results are shown in Table 1. The extraction efficiency ranged from 84.82% to 94.11%, which indicated that the considerable variation in extraction efficiency depending on different extraction conditions. The regression coefficients of the second-order polynomial equation and the results of ANOVA for the krill oil extraction efficiency were presented in Table 2. The higher *F*-value (101.15) was associated with lower *p*-value (*p* < 0.001) for the model, which implied that the model was valid. The determination coefficients (*R*^2^) of this model (0.9924) was closer to 1, which suggested a good correlation between experimental results and the model. The results of error analysis stated that the lack of fit was insignificant (*p* > 0.05), which means that the model was adequate to predict the extraction efficiency with different combination of values of the variables. The predicted *R*^2^ of 0.9426 was in reasonable agreement with the adjusted *R*^2^ of 0.9826. The model also had a low value of CV (0.40), which indicated that it was reproducible and had high degree of precision and good reliability of conducted experiments. The model's predicted residual sum of squares (PRESS), which was used to measure the matching of each point in the design with the model, was 6.89. The value of adequate precision (signal to noise ratio) was 32.389, which suggested the best fit of the developed model. This model could be used to navigate the design space. In conclusion, the model equation was reproducible, credible and adequate for predicting the krill lipid recovery rate under any combination of values of the variables.

**Table 2:** ANOVA analysis and statistical parameters of the model

Source	Sum of squares	df	Mean square	*F* value	Prob > *F*
Model	119.03	9	13.23	101.15	<0.0001
*A*-stirring speed	6.53	1	6.53	49.97	0.0002
*B*-dynamic extraction time	15.04	1	15.04	115.05	<0.0001
*C*-temperature	77.88	1	77.88	595.61	<0.0001
*AB*	0.21	1	0.21	1.58	0.2486
*AC*	0.23	1	0.23	1.76	0.2260
*BC*	0.71	1	0.71	5.40	0.0532
*A* ^2^	1.66	1	1.66	12.71	0.0092
*B* ^2^	1.88	1	1.88	14.38	0.0068
*C* ^2^	13.50	1	13.50	103.27	<0.0001
Residual	0.92	7	0.13		
Lack of fit	0.38	3	0.13	0.94	0.5010
Pure error	0.54	4	0.13		
Cor total	119.94	16			
*R* ^2^	0.9924		Adj-*R*^2^	0.9826	
CV%	0.40		Pred-*R*^2^	0.9426	
PRESS	6.89		Adeq precisior	32.389	

Further, multiple regression analysis was employed on the experimental data, and it was shown that the predicted extraction efficiency of krill oil (*Y*) could be calculated according to the below second-order polynomial model: *Y* = 92.39 + 0.90*X*_1_ + 1.37*X*_2_ + 3.12*X*_3_ − 0.23*X*_1_*X*_2_ − 0.24*X*_1_*X*_3_ − 0.42*X*_2_*X*_3_ − 0.63*X*_1_^2^ − 0.67*X*_2_^2^ − 1.79*X*_3_^2^, where *X*_1_, *X*_2_, and *X*_3_ are means of the coded factors of the stirring speed, dynamic extraction time and extraction temperature, respectively.

**Fig. 2:**
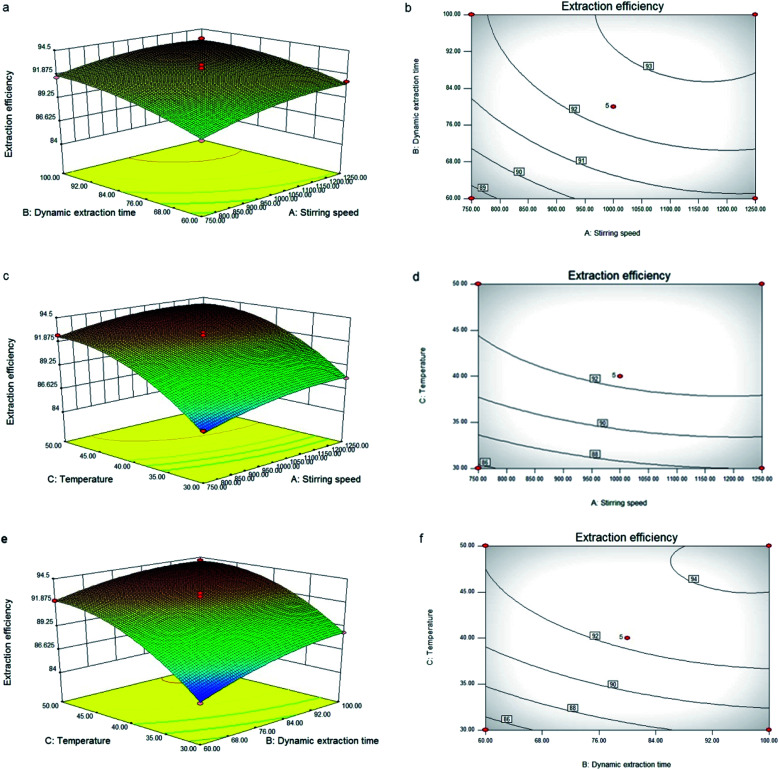
Response surface plots (a, c and e) and contour plots (b, d and f) of the oil yield affected by stirring speed (A), dynamic extraction time (B) and temperature (C).

The three-dimensional (3D) response surface and two-dimensional (2D) contour plots that are the graphical representations of regression equation obtained from the calculated response surface are shown in Fig. 2. The interactions between two variables and their optimum ranges can be seen. All the mutual interactions between the test variables were found to be insignificant (*p* > 0.05). The predicted extraction conditions for this mode were as follows: stirring speed 1030.20 rpm, dynamic extraction time 91.37 min, extraction temperature 46.8 °C. Under the optimal conditions, the maximum extraction efficiency was 94.15%. In consideration of the convenience for practical experiment, the optimum test variables were determined as follows: stirring speed 1030 rpm, dynamic extraction time 90 min, extraction temperature 47 °C. Under the new conditions, the average extraction efficiency was 93.77 ± 0.92%, which was reasonable as compared to the predicted value (94.15%). This result indicated that the response model was accurate and valid.

### 3.2. Extraction efficiency of krill oil with different extraction methods

**Fig. 3:**
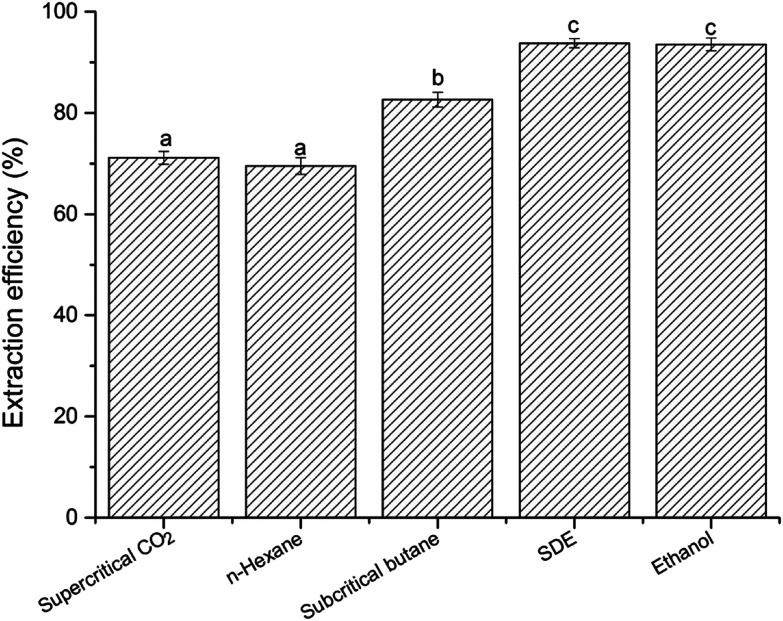
Extraction efficiency of krill oil by different extraction methods. Different letters in the same row indicate significant differences (Tukey's test, *p* < 0.05). SDE, subcritical dimethyl ether.

The lipid extraction efficiency of different extraction methods ranged from 69.53 ± 1.62 to 93.77 ± 0.92%, as shown in Fig. 3. The order of extraction efficiency with different extraction methods was as follow: *n*-hexane (69.53 ± 1.62%) < supercritical CO_2_ (71.15 ± 1.27%) < subcritical *n*-butane (82.64 ± 1.48%) < ethanol (93.55 ± 1.26%) < SDE (93.77 ± 0.92%). There was no significant difference (*p* > 0.05) for extraction efficiency between the *n*-hexane and supercritical CO_2_ extraction. The lower extraction efficiency might be associated with their weaker solubility for polar phospholipid components. Both of SDE and ethanol showed higher (*p* > 0.05) extraction efficiency than other extraction methods. According to previous study, DE was a strong solvent for both neutral and polar lipids.^20^ Therefore, SDE can extract relatively all krill oil and display higher extraction efficiency. Our findings were consistent with the result described by Kanda, Li, Ikehara and Yasumoto-Hirose,^19^ which demonstrated that SDE could directly extract lipids from high-moisture microalgae with an extraction yield almost equivalent to that of the Bligh–Dyer's method. In addition, it was found that it was found that SDE also has higher extraction efficiency for water was around 85%. So, it indicated that krill oil and water could be co-extracted from wet Antarctic krill using SDE extraction method. However, other extraction methods were just suitable for extraction krill oil from dried krill meal. Those results demonstrated that the processing procedure of krill oil would be simplified due to free-of the drying step, and meanwhile reducing the overall cost. SDE extraction technique could be applied in food processing areas for extraction organic constituents and dewatering in future.

### 3.3. Chemical properties of krill oil

The acid value (AV) and peroxide value (POV) were usually used to reflect the quality of the lipids. As shown in Table 3, the AV of krill oil obtained by SDE and supercritical CO_2_ presented higher AV than other extraction methods. The high AV of krill oil extracted by SDE was possibly attributed to long-time defrosting (50 min) using running water, which gave rise to hydrolysis of lipids by lipase. Krill oil extracted by supercritical CO_2_ tended to have low levels of antioxidants and high levels of neutral lipids including free fatty acids,^34^ resulting in the relatively higher AV and POV. The krill oil extracted by SDE and subcritical *n*-butane were performed at relatively vacuum environment and desolventizing temperature, which contributed to obtain krill oil of lower POV (1.46 ± 0.26 and 2.14 ± 0.25 mmol kg^−1^, respectively). This result was in agreement with our previous research,^25^ which confirmed that SDE was an advanced technology for obtaining oil of lower POV. The POV of krill oil extracted by *n*-hexane and ethanol was similar (*p* > 0.05) and had the highest POV. The iodine value was used to reflect the unsaturation of krill oil. There were similar results (*p* > 0.05) between the iodine values of lipids obtained by ethanol and SDE. The non-saponifiable matters were used to measure the amount of non-glyceride-soluble matters. The content of non-saponifiable matters in krill oil extracted using ethanol and SDE had no significant differences (*p* > 0.05) and they presented lower level than other methods. Additionally, DE (−24.8 °C) has a lower boiling point than *n*-butane (−0.5 °C), *n*-hexane (69 °C) and ethanol (78 °C), which indicated that DE was easier to be removed from oil and has relatively small residual amount.

**Table 3:** Chemical properties of krill oil by different extraction methods^a^

Property	Supercritical CO_2_	*n*-Hexane	Subcritical *n*-butane	SDE	Ethanol
Acid value (mg KOH per g)	26.36 ± 0.44^d^	18.73 ± 0.43^a^	20.60 ± 0.68^b^	33.33 ± 0.56^e^	23.11 ± 1.05^c^
Peroxide value (mmol kg^−1^)	2.68 ± 0.13^c^	3.13 ± 0.18^d^	2.14 ± 0.25^b^	1.46 ± 0.26^a^	3.15 ± 0.22^d^
Iodine value (×10^−2^ g I_2_ per g)	100.84 ± 1.46^a^	107.69 ± 0.89^b^	112.48 ± 1.38^c^	117.31 ± 2.00^d^	120 ± 1.60^d^
Non-saponification matter (%)	4.66 ± 0.10^c^	4.44 ± 0.13^b^	4.57 ± 0.09^c^	4.04 ± 0.18^a^	3.98 ± 0.14^a^

a Different letters in the same row indicate significant differences (Tukey's test, *p* < 0.05). SDE, subcritical dimethyl ether.

### 3.4. PC and PE contents of extracted krill oil

The contents of two types phospholipids (PLs) as percentage of krill oil mass were shown in Table 4. It was noted that the content of PC was not detected and produced the lowest PE content (4.04% ± 0.06%) in the krill oil extracted using supercritical CO_2_. It was stated that pure CO_2_ is unable to interact with either polar lipids or neutral lipids, which means that it is not an effective solvent for extraction of PC and PE.^17^ In the mass, subcritical *n*-butane can obtain more PC and PE than *n*-hexane and supercritical CO_2_. This result displayed a similar trend as reported by Sun, *et al.*^18^ Moreover, the content of PC of krill oil extracted by SDE (33.95 ± 0.65%) was just below that extracted by ethanol (38.8 ± 0.30%), but the content of PE (11.67 ± 0.23%) was superior than that extracted by other methods. This result further confirmed the results described by Catchpole, Tallon, Eltringham, Grey, Fenton, Vagi, *et al.*,^35^ who concluded that DE has a high degree of solubility for polar lipid and is an exceptional solvent for all classes of PLs. In addition, it has been reported that the solubility of phospholipids (soy lecithin) in DE increased around 10% with water as a co-solvent at 333 K.^16^ This means that the water in the wet Antarctic krill might be a co-solvent for DE, which is beneficial to the extraction of PLs. Therefore, DE as a safe solvent might be selected to extraction of polar lipid for the practical industrial applications.

**Table 4:** PC, PE, astaxanthin, α-tocopherols and vitamin A contents of krill oil extracted by different extraction methods^a^

	Supercritical CO_2_	*n*-Hexane	Subcritical *n*-butane	SDE	Ethanol
PC (%)	ND	16.05 ± 0.66^a^	25.14 ± 0.77^b^	33.95 ± 0.65^c^	38.80 ± 0.30^d^
PE (%)	4.04 ± 0.06^a^	5.97 ± 0.12^b^	8.61 ± 0.07^c^	11.67 ± 0.23^e^	10.47 ± 0.12^d^
Astaxanthin (mg kg^−1^)	80.55 ± 9.97^a^	122.75 ± 8.93^b^	127.79 ± 6.86^b^	220.06 ± 7.57^c^	236.11 ± 7.16^d^
α-Tocopherol (mg/100 g)	33.37 ± 0.51^a^	49.98 ± 1.41^d^	49.87 ± 1.50^d^	39.89 ± 0.70^c^	35.49 ± 0.95^b^
Vitamin A (mg kg^−1^)	74.40 ± 0.94^c^	92.04 ± 2.37^d^	75.91 ± 2.20^c^	70.25 ± 1.19^b^	63.91 ± 1.21^a^

a Different letters in the same row indicate significant differences (Tukey's test, *p* < 0.05). Abbreviations are: PC, phosphatidylcholine; PE, phosphatidylethanolamine; SDE, subcritical dimethyl ether; ND, not detected.

### 3.5. Fatty acids profile analysis

Table 5 lists the composition and relative contents of fatty acids in extracted krill oil with different extraction methods. It was obvious that krill fatty acids ranged from 12 to 22 carbons in length. Having C14:0, C16:0, C16:1, C18:1, C20:5, and C22:6 as the major fatty acids, this was in agreement with the previous results reported by Gigliotti, Davenport, Beamer, Tou and Jaczynski,^12^ and Yin, *et al.*^15^ It was worth noting that the trend of EPA and DHA contents was consistent with PLs (PC and PE) contents in the extracted krill oil. This result was similar to previous reports by Gigliotti, Davenport, Beamer, Tou and Jaczynski,^12^ and Xie *et al.*,^13^ who demonstrated that EPA and DHA had higher association with PLs. Therefore, krill oil extracted by SDE and ethanol showed higher amount of EPA and DHA (24.29 ± 0.10 and 25.73 ± 0.08%, respectively) than other methods. Moreover, krill oil extracted with ethanol and SDE contained more PUFAs than that extracted by other methods. As for the SFA and MUFA contents of the krill oil extracted, supercritical CO_2_, *n*-hexane, subcritical *n*-butane displayed higher than those by SDE and ethanol.

**Table 5:** Fatty acid compositions (area%) of krill oil extracted by different extraction methods^a^

Fatty acids	Supercritical CO_2_	*n*-Hexane	Subcritical *n*-butane	SDE	Ethanol
C12:0	0.65 ± 0.03^d^	0.56 ± 0.01^c^	0.45 ± 0.03^b^	0.46 ± 0.04^b^	0.38 ± 0.01^a^
C13:0	0.31 ± 0.01^c^	0.28 ± 0.01^c^	0.21 ± 0.02^b^	0.23 ± 0.02^b^	0.19 ± 0.02^a^
C14:0	9.32 ± 0.21^e^	8.85 ± 0.05^c^	9.00 ± 0.03^d^	6.98 ± 0.01^a^	7.33 ± 0.04^b^
C14:1	0.50 ± 0.02^d^	0.46 ± 0.02^c^	0.34 ± 0.01 ^ab^	0.35 ± 0.01^b^	0.31 ± 0.01^a^
C15:0	1.39 ± 0.01^e^	1.31 ± 0.01^d^	1.03 ± 0.01^a^	1.20 ± 0.02^c^	1.13 ± 0.01^b^
C16:0	16.20 ± 0.01^c^	16.35 ± 0.03^c^	16.43 ± 0.04^c^	14.95 ± 0.06^a^	15.58 ± 0.32 ^ab^
C16:1	12.07 ± 0.11^d^	11.57 ± 0.06^c^	10.62 ± 0.03^b^	9.51 ± 0.04^a^	9.49 ± 0.05^a^
C17:0	0.85 ± 0.01^b^	0.84 ± 0.01^b^	0.62 ± 0.00^a^	1.36 ± 0.02^c^	0.85 ± 0.01^b^
C17:1	0.91 ± 0.03^c^	0.88 ± 0.00^bc^	0.80 ± 0.02^a^	0.98 ± 0.01^d^	0.86 ± 0.01^b^
C18:0	3.56 ± 0.01^d^	3.63 ± 0.02^e^	3.03 ± 0.01^b^	3.35 ± 0.01^c^	2.91 ± 0.02^a^
C18:1 (*n*-9)	18.10 ± 0.18^d^	17.49 ± 0.13^c^	17.57 ± 0.10^c^	15.25 ± 0.05^b^	14.80 ± 0.06^a^
C18:1 (*n*-7)	10.37 ± 0.19^c^	10.22 ± 0.07^c^	9.09 ± 0.02^a^	9.34 ± 0.02^b^	9.53 ± 0.05^b^
C18:2 (*n*-6)	4.18 ± 0.03^d^	4.05 ± 0.02^c^	3.55 ± 0.04^a^	3.99 ± 0.02^c^	3.77 ± 0.02^b^
C18:3 (*n*-6)	0.60 ± 0.00^d^	0.58 ± 0.01^c^	0.47 ± 0.01^a^	0.56 ± 0.01^c^	0.49 ± 0.01^b^
C18:3 (*n*-3)	2.37 ± 0.01^c^	2.26 ± 0.01^b^	2.00 ± 0.01^a^	2.39 ± 0.01^c^	2.26 ± 0.01^b^
C20:1 (*n*-9)	1.76 ± 0.01^c^	1.77 ± 0.05^c^	1.81 ± 0.05^c^	1.62 ± 0.01^b^	1.36 ± 0.01^a^
C20:3 (*n*-6)	0.13 ± 0.01^a^	0.15 ± 0.00^b^	0.18 ± 0.01^c^	0.18 ± 0.01^c^	0.17 ± 0.03^bc^
C20:4 (*n*-6)	0.54 ± 0.02^a^	0.50 ± 0.00^a^	0.59 ± 0.01^b^	0.82 ± 0.03^d^	0.71 ± 0.01^c^
C20:4 (*n*-3)	0.52 ± 0.05^a^	0.62 ± 0.00^b^	0.67 ± 0.00^b^	0.81 ± 0.04^c^	0.80 ± 0.01^c^
C20:5 (*n*-3)	11.18 ± 0.07^a^	11.92 ± 0.14^b^	13.27 ± 0.03^c^	16.38 ± 0.05^d^	17.83 ± 0.07^e^
C22:1	0.43 ± 0.02^a^	0.65 ± 0.00^b^	0.98 ± 0.05^c^	0.94 ± 0.02^c^	0.92 ± 0.00^c^
C22:5 (*n*-3)	0.27 ± 0.00^a^	0.28 ± 0.00^a^	0.43 ± 0.01^b^	0.44 ± 0.02^b^	0.46 ± 0.01^b^
C22:6 (*n*-3)	3.76 ± 0.05^a^	4.79 ± 0.18^b^	6.91 ± 0.12^c^	7.91 ± 0.07^d^	7.89 ± 0.04^d^
EPA + DHA	14.95 ± 0.05^a^	16.70 ± 0.06^b^	20.18 ± 0.09^c^	24.29 ± 0.10^d^	25.73 ± 0.08^e^
∑SFA	32.29 ± 0.03^d^	31.82 ± 0.11^c^	30.78 ± 0.06^b^	28.53 ± 0.07^a^	28.35 ± 0.25^a^
∑MUFA	44.15 ± 0.11^e^	43.04 ± 0.14^d^	41.23 ± 0.07^c^	38.00 ± 0.06^b^	37.26 ± 0.17^a^
∑PUFA	23.55 ± 0.09^a^	25.14 ± 0.06^b^	28.08 ± 0.04^c^	33.49 ± 0.06^d^	34.39 ± 0.11^e^

a Different letters in the same row indicate significant differences (Tukey's test, *p* < 0.05). Abbreviations are: SDE, subcritical dimethyl ether; EPA, eicosapentaenoic acid; DHA, docosahexaenoic acid; SFA, saturated fatty acids; MUFA, monounsaturated fatty acids; PUFA, polyunsaturated fatty acids.

### 3.6. Astaxanthin content of extracted krill oil

Astaxanthin is one of polar carotenoids with the oxygen-bearing functional groups such as –OH and =O.^35^ Therefore, astaxanthin has higher antioxidant capacity as compared with other carotenoids. Table 4 showed the astaxanthin content of the krill oil extracted with different extraction methods. The lowest astaxanthin amount (80.55 ± 9.97 mg kg^−1^) was observed in krill oil extracted by supercritical CO_2_. This result was similar to previous study that supercritical CO_2_ was not the most suitable solvent for extraction of astaxanthin, and it was found that the highest yield of astaxanthin was around 8.62 mg/100 g at 25 MPa and 45 °C.^28^ The solubility of astaxanthin in supercritical CO_2_ is limited because it is a large molecule and polar carotenoid.^36^ The astaxanthin contents extracted by *n*-hexane and subcritical *n*-butane (122.75 ± 8.93 and 127.79 ± 6.86 mg kg^−1^, respectively) showed no significant difference (*p* > 0.05), accounting for only half of those extracted by SDE and ethanol. This result was in agreement with the previous report by Xie, *et al.*,^13^ who reported that the astaxanthin content of krill extracted by *n*-hexane was similar to that by subcritical *n*-butane and produced approximately 100 mg kg^−1^ of astaxanthin content. The yield of astaxanthin using SDE (220.06 ± 7.57 mg kg^−1^) was slightly inferior to ethanol (236.11 ± 7.17 mg kg^−1^). It can be concluded that SDE was an effective method to obtain higher quality of krill oil owing to its higher solubility for astaxanthin. Therefore, further development on the extraction of carotenoids by SDE is feasible.

### 3.7. Tocopherols and vitamin A content of extracted krill oil

It was found that the γ-tocopherol of krill oil obtained by the five different extraction methods was not detected. It has been reported that tocopherol of krill oil was mostly present in the form of a-tocopherol, theγ-tocopherol was present in very small amount. It possibly means that the γ-tocopherol content of raw Antarctic krill used in this study was too low to determine. α-Tocopherol and vitamin A contents of krill oil extracted by different methods are shown in Table 4. As can be seen, krill oil extracted by subcritical *n*-butane and *n*-hexane showed no significant differences (*p* > 0.05) in the contents of a-tocopherol and resulted in higher concentration of a-tocopherol (49.87 ± 1.50 and 49.98 ± 1.41 mg/100 g, respectively) than other methods. The content of a-tocopherol (39.89 ± 0.70 mg/100 g) extracted by SDE was at medium level and only slightly lower than that extracted by subcritical *n*-butane and *n*-hexane. Numerically, the order of vitamin A content of krill oil extracted by different extraction methods is similar to above-mentioned content of a-tocopherol. It was obvious that the contents of vitamin A and a-tocopherol for the krill oil extracted by ethanol were the lowest. This was possibly attributed to lower solubility of the type of neutral lipids that did not contain fatty acids (hydrocarbons, sterols, ketones, carotenes and chlorophylls) in polar ethanol. Similar to ethanol extraction, SDE was not the most efficient extraction method for vitamin A, but it is an effective method for extracting much more PC, PE and astaxanthin (as mentioned in Sections 3.4 and 3.6).

## 4. Conclusion

This work displayed the feasibility of extraction of krill oil from wet Antarctic krill by SDE. And the optimal extraction conditions for the SDE extraction of krill oil were obtained based on statistical methodology and Box–Behnken Response Surface design. Moreover, the comparison and analysis on the chemical properties and nutritive quality of krill oil extracted by SDE and other four extraction methods were conducted. Results showed that the quality of krill oil extracted by SDE was partially similar to that extracted by ethanol and superior to that extracted by *n*-hexane, supercritical CO_2_ and subcritical *n*-butane. For example, SDE extraction of krill oil obtained with the lowest peroxide value and much more content of PC, PE, PUFA and astaxanthin. Besides, it is worth noting that SDE can directly extract the organic constituents and moisture from the natural feedstock. Therefore, SDE technique could be hopefully applied in food and pharmaceutical industry and seen great commercial value.

## Conflicts of interest

The authors declare that they have no conflicts interests.

## Supplementary Material
